# Body Composition Changes and Associations in Infants and Mothers During the First Year: Insights from a Pilot Study of the Baby-bod Project

**DOI:** 10.3390/children12010097

**Published:** 2025-01-16

**Authors:** Manoja P. Herath, Kiran D. K. Ahuja, Jeffrey M. Beckett, Sisitha Jayasinghe, Nuala M. Byrne, Andrew P. Hills

**Affiliations:** School of Health Sciences, College of Health and Medicine, University of Tasmania, Launceston, TAS 7248, Australia; manoja.herath@utas.edu.au (M.P.H.); kiran.ahuja@utas.edu.au (K.D.K.A.); jeffrey.beckett@utas.edu.au (J.M.B.); sisitha.jayasinghe@utas.edu.au (S.J.); nuala.byrne@utas.edu.au (N.M.B.)

**Keywords:** infant body composition, maternal body composition, postpartum period, prepregnancy body mass index, prepregnancy overweight and obesity

## Abstract

Background: The period following childbirth is marked by dynamic changes in maternal physiology and the growth trajectory of the newborn. We aimed to elucidate the changes and associations in body composition of infants and their mothers during the first year postpartum. Methods: This pilot study assessed infant body composition using the PEA POD air displacement plethysmography (ADP) system (birth–6 months) and deuterium dilution (9–12 months). Maternal body composition was assessed using the BOD POD ADP system at 12 months postpartum. Mothers were grouped by prepregnancy body mass index (BMI) <25 kg/m^2^ (lean) or ≥25 kg/m^2^ (overweight/obese: OW/OB), and data were analysed using linear regression. Results: Twenty-nine infant–mother pairs were assessed. Infant percent fat mass (%FM) increased from birth to 6 months (9.3% vs. 24.2%; *p* < 0.001) and then gradually declined. At birth and 3 months, %FM was significantly higher in infants born to OW/OB mothers compared to their lean counterparts. A significant positive association (β = 0.3; *p* = 0.040) was observed between maternal %FM and infant %FM at 1 year post-delivery after controlling for the mother’s prepregnancy BMI. Conclusions: Infants born to OW/OB mothers have increased %FM at birth and 3 months, which may have consequences for their health throughout childhood and into adulthood. Moreover, maternal prepregnancy BMI is a significant predictor of maternal postpartum weight status and body composition and impacts the relationship between maternal and infant body composition at 12 months postpartum. While the findings of our pilot study underscore the importance of encouraging women of childbearing age to maintain a healthy BMI before conception, further research is needed to substantiate these results.

## 1. Background

The first 1000 days of life represent a critical window of growth and development that has the potential to shape lifelong health [1,2]. During this period, a series of complex physiological adaptations occur in both the infant and the mother, impacting the dynamics of body composition [3,4]. Excess fat mass (FM) accrual during infancy has been found to track into childhood and then adulthood, leading to potential health consequences associated with obesity [5,6]. Similarly, postpartum weight retention in mothers is associated with preferential accumulation of FM, especially in the visceral compartment, and is a strong determinant of later-life obesity-related chronic diseases [7,8].

Previous research to identify determinants of excess FM during early life has largely explored associations with maternal weight status based on body mass index (BMI). The impact of maternal BMI on the growth and development of offspring in the early stages of life is influenced by genetic and epigenetic predisposition and obesogenic environmental exposures [9]. Several previous studies have indicated a positive association between maternal prepregnancy BMI and FM in infants [10,11,12]. Further, a systematic review and meta-analysis by Castillo-Laura et al. [13] identified greater standardised mean differences in FM and percent fat mass (%FM) in children born to overweight/obese (OW/OB; BMI ≥ 25 kg/m^2^) mothers compared to those of lean mothers (BMI < 25 kg/m^2^).

Objective assessment of body composition is strongly encouraged as BMI does not consider age, muscle mass, bone structure, or distribution of body fat [14]. However, to date, only a few studies have investigated infant–mother body composition associations. In a study with 63 infant–mother dyads, Butte et al. [15] found that infant birthweight positively correlated with maternal prepregnancy FM but not with a gestational gain of FM or postpartum FM. In another study (n = 30), Pomeroy et al. [16] reported that neither maternal FM, fat-free mass (FFM), or %FM measured at 28–32 weeks of gestation was significantly associated with infant FM, FFM, or %FM at four months of age. To the best of our knowledge, no published study has examined infant–mother body composition associations to 1 year postpartum. We employed data from a pilot study of infant–mother dyads conducted in Tasmania, where ~55% of women enter pregnancy with an OW/OB prepregnancy BMI [17], to explore the following:

- Changes in body composition of infants from birth to 1 year of age;
- Changes in weight and BMI in mothers from preconception to 1 year postpartum to determine the association between prepregnancy BMI and body composition at the 1-year mark;
- Associations between body composition of infants and mothers at 1 year postpartum.

## 2. Methods

### 2.1. Participant Recruitment

The Baby-bod study [18,19] was a prospective cohort study and the Australian arm of the Multi-center Infant Body Composition Reference Study (MIBCRS) [20] to determine body composition references for 0- to 2-year-old infants. Inclusion criteria for this study were singleton term pregnancy (gestational age at birth between 37^+0^ and 41^+6^ weeks), mothers age ≥ 18 years, and ability to speak and understand English. Exclusion criteria were newborns with a congenital anomaly or admitted to the neonatal intensive care unit, mothers with difficult birthing experience, or inability to negotiate the informed consent process. Body composition assessments of infants from birth to 6 months of age were conducted at the Launceston General Hospital, Tasmania, between September 2017 and October 2019. Follow-up body composition assessments were conducted when the infants reached 9 and 12 months of age, with maternal body composition evaluated during the 12-month visit. The 9- and 12-month assessments took place at the University of Tasmania (Newnham Campus) between August 2019 and March 2020. All research procedures and protocols were approved by the Human Research Ethics Committee of Tasmania (reference no: H0016117).

### 2.2. Data Collection

#### 2.2.1. Infants

Infant body composition was evaluated from birth to one year of age at three-month intervals. The PEA POD ADP system (COSMED USA Inc., Concord, CA, USA; software version 3.5.0) was employed to evaluate infant body composition at birth, 3 months, and 6 months of age. The operating procedures of the PEA POD have been described in detail elsewhere [21,22]. In brief, infant demographics, including length measured using an infantometer (Seca 417, Hamburg, Germany), were entered into the PEA POD software. The PEA POD software uses measured weight (using the integrated weighing scale) and volume with the density model by Fomon et al. [23] to calculate %FM.

As the PEA POD only accommodates infants to ~6 months (8–10 kg), the deuterium dilution technique was used to assess infant body composition at 9 and 12 months. The principles and procedures of the deuterium dilution technique have been described in detail previously [24]. Briefly, a sample of saliva was obtained from the infant using a sterile cotton ball and plastic forceps, and then one gram of undiluted deuterium oxide (D_2_O 99.8%) was administered using a sterile syringe. Post-dose saliva samples were collected at 2.5 and 3 h after the dose administration using sterile cotton balls and plastic forceps. The collected samples were stored at −80 °C until analysis. D_2_O concentration in the samples was analysed using the Agilent 4500 FTIR portable spectroscopy instrument (Agilent Technologies, Inc., Santa Clara, CA, USA) [25]. The dilution principle (V_2_ = C_1_V_1_/C_2_) and hydration factors of infants from Fomon et al. [23] were used to calculate %FM.

#### 2.2.2. Mothers

At the initial assessment, mothers self-reported their prepregnancy weight and birth-related information (e.g., gestational age). In addition, height was measured to the nearest millimetre using a stadiometer (SECA Corp., Hamburg, Germany). At each subsequent visit, weight was measured to the nearest gram using a digital scale (SECA Corp. Hamburg, Germany) and maternal BMI was calculated as body weight (kg)/height^2^ (m). Body composition at 12 months postpartum was assessed using the BOD POD ADP system (COSMED USA Inc., Concord, CA, USA; software version v-5.3) with thoracic gas volume measured using a disposable tube and filter. Participants were asked to avoid food or exercise for 2 h, empty the bladder prior to the assessment, and wear minimal, form-fitting clothing and a swim cap for volume measurement. During the assessment, basic demographic information (e.g., date of birth) and height were entered into the BOD POD computer system. Body mass was measured using the integrated scale, and body volume was measured while the participant was sitting inside the BOD POD chamber. Body density calculated from body weight and volume and measured thoracic gas volume are used in the densitometric equation by Siri [26] to calculate %FM.

### 2.3. Statistical Analysis

All analyses were performed using R Project for Statistical Computing (version 4.2.0, Vienna, Austria) [27]. Descriptive variables are expressed using mean and standard deviation (SD) or number and percentage (%). Linear regression was used to assess the differences in measures between time points and infant–mother body composition associations. kNN imputation was used to handle missing data, which accounted for 8% of the dataset. An interaction term with maternal prepregnancy BMI was added to assess the difference between the two groups: OW/OB (prepregnancy BMI ≥ 25 kg/m^2^) and lean (prepregnancy BMI < 25 kg/m^2^). All hypothesis tests were two-sided, and statistical significance was set at *p* < 0.05.

## 3. Results

### 3.1. Participants

Twenty-nine infant–mother dyads participated in assessments from birth to 1 year. Mothers were predominantly Caucasian (93%), and their mean ± SD age at delivery was 29.9 ± 4.4 years. Out of the 29 mothers, 18 (62%) had an OW/OB prepregnancy BMI, while the other 11 (38%) had a lean prepregnancy BMI. The mean ± SD weight and length of the infants at birth were 3.3 ± 0.5 kg and 49.69 ± 2.1, respectively, and the majority (76%) were males (Table 1). All the infants were breastfed up to 6 months, and a majority (64%) continued to breastfeed until 12 months.

### 3.2. Changes in Body Composition of Infants from Birth to 1 Year

**Figure 1:**
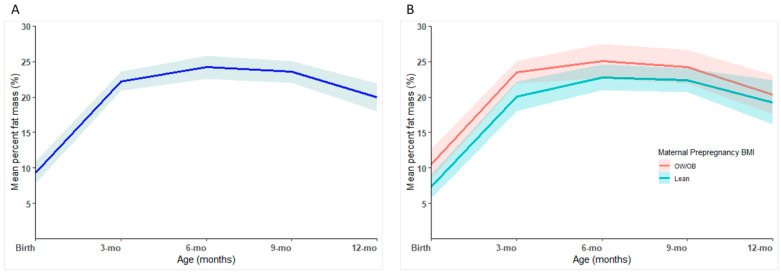
(**A**) Changes in mean percent fat mass (%) in infants from birth to 1 year. (**B**) Changes in mean percent fat mass (%) in infants from birth to 1 year according to maternal prepregnancy body mass index (BMI). ‘OW/OB’: overweight/obese is BMI ≥ 25 kg/m^2^, and ‘Lean’ is BMI < 25 kg/m^2^; shaded areas in each graph show the 95% confidence interval.

Infant %FM more than doubled from birth to 3 months (9.3% vs. 22.2%; *p* < 0.001) and further increased until ~6 months of age (24.2%) and gradually declined afterwards (Figure 1). Infants born to OW/OB mothers had significantly higher %FM at birth (10.5% vs. 7.4%; *p* = 0.046) and 3 months (23.5% vs. 20.1%; *p* = 0.033) than those of lean mothers, but the difference was not significant at subsequent time-points.

### 3.3. Changes in Weight and BMI of Mothers from Preconception to 1 Year Postpartum

**Figure 2:**
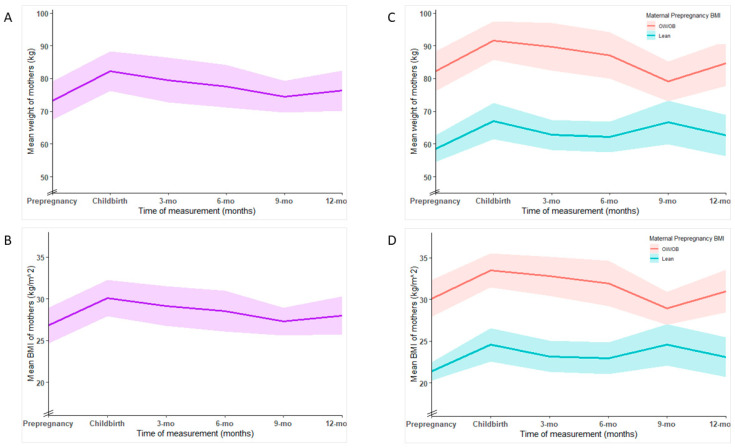
(**A**) Changes in mean weight (kg) of mothers from prepregnancy to 1 year postpartum. (**B**) Changes in mean body mass index (BMI, kg/m^2^) of mothers from prepregnancy to 1 year postpartum. (**C**) Changes in mean weight (kg) of mothers from prepregnancy to 1 year postpartum according to maternal prepregnancy BMI category. (**D**) Changes in mean BMI (kg/m^2^) of mothers from prepregnancy to 1 year postpartum according to maternal prepregnancy BMI category. ‘OW/OB’: overweight/obese is BMI ≥ 25 kg/m^2^, and ‘Lean’ is BMI < 25 kg/m^2^; shaded areas in each graph show the 95% confidence interval.

On average, mothers experienced a weight gain of 9 kg from prepregnancy to childbirth (73.3 kg vs. 82.3 kg; *p* = 0.035), and their mean BMI increased by 3.3 units (26.8 kg/m^2^ vs. 30.1 kg/m^2^; *p* = 0.031). Mother’s mean postpartum weight and BMI gradually decreased until 9 months and slightly increased afterwards (Figure 2). Postpartum weight retention at 3 and 6 months was 6.2 kg and 4.3 kg, respectively, and decreased to a low of 1.1 kg at 9 months and increased again to 3 kg at 12 months. However, their mean weight and BMI at 3 months and subsequent assessments were not significantly different (*p* > 0.1 for all) from the prepregnancy parameters. Compared to lean mothers, OW/OB mothers had significantly higher mean weight and BMI throughout the postpartum period. The difference in mean weight between the two groups was >20 kg (*p* < 0.001) throughout (except at 9 months, 12.5 kg; *p* = 0.005). Similarly, there was a BMI difference of 8–9 units (*p* < 0.001) through this period, the exception again being at 9 months (a difference of 4.4 units; *p* = 0.007).

### 3.4. Maternal Postpartum Body Composition and Its Relationship with Prepregnancy BMI

**Figure 3:**
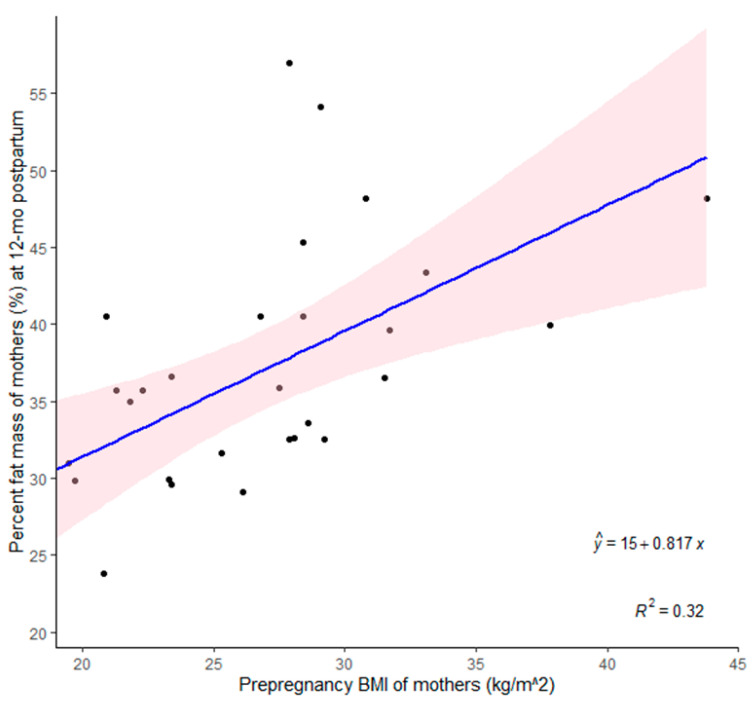
Association between prepregnancy body mass index (BMI) and percent fat mass (%) of mothers at 1 year postpartum. Dots represent individual data points, the blue line denotes the regression line, and the shaded area represents the 95% confidence interval.

The mean ± SD maternal %FM at 12 months postpartum was 36.9 ± 8.1%. Mothers’ prepregnancy BMI explained a 32% variation in their %FM at 12 months postpartum. For every unit increase in prepregnancy BMI, %FM at 12 months postpartum increased by 0.8% (*p* = 0.001; Figure 3). At 12 months postpartum, mothers’ BMI explained 81% variation in maternal %FM. For every unit increase in BMI, %FM increased by 1.2% (*p* < 0.001).

### 3.5. Associations Between Body Composition of Infants and Mothers at 12 Months Post-Childbirth

Although the association of %FM between infants and mothers at the 12-month post-childbirth mark did not reach statistical significance (β = 0.2; *p* = 0.083), it became significant when accounting for maternal prepregnancy BMI (β = 0.3; *p* = 0.040; Table 2).

## 4. Discussion

This study assessed body composition changes and associations in Tasmanian infants and mothers during the first year post-childbirth. Infant %FM increased rapidly from birth to 3 months, peaked at 6 months, and gradually declined thereafter. %FM at birth and 3 months was significantly higher in infants born to OW/OB mothers than those born to lean mothers. While mothers’ postpartum weight and BMI were not significantly different to their prepregnancy parameters from 3 months onwards, postpartum weight retention reduced from ~6 kg at 3 months to ~3 kg at 12 months. However, mothers with OW/OB prepregnancy BMI consistently exhibited significantly higher postpartum weight and BMI in comparison to lean mothers. At 12 months postpartum, the association between maternal %FM and infant %FM showed a positive trend and became statistically significant after adjusting for prepregnancy BMI.

This research contributes to the growing body of knowledge encompassing maternal-infant body composition dynamics during the postpartum period, and our findings may have several implications. We observed that influences of maternal weight status on %FM levels in infants persisted from birth to 3 months, the period where rapid growth in body fat occurs. This is particularly important as there can be potential long-term health implications of increases in infant %FM during these critical early months. Future research should investigate whether early differences in body fat levels have lasting effects on the health and development of infants. Moreover, although postpartum weight retention decreased over time, it is important to note that OW/OB mothers consistently had significantly higher weight and BMI levels compared to their lean counterparts throughout the postpartum period. While this observation may not come as a surprise, it underscores the lasting impact of maternal prepregnancy BMI on postpartum body composition outcomes. Our finding that maternal %FM at 12 months postpartum has an independent impact on infant %FM when adjusted for maternal prepregnancy BMI underscores the importance of considering both maternal postpartum %FM and prepregnancy BMI when studying their effects on infant %FM.

The trajectory of adiposity development during the first year of life observed in this Tasmanian cohort corroborates published body composition reference data for healthy-term-born infants [20,28,29,30] that infant %FM increases from 0 to 6 months and declines between 6 and 12 months. This peaking of adiposity around 6 months of age has been described as an evolutionary adaptation [31]. Six months of age is usually the time infants start weaning, and having enough energy stored around this period could be a mechanism to prepare the infant for the unstable weaning period. Furthermore, our findings agree with several studies [10,11,32,33,34] that reported increased early-life adiposity in the offspring of OW/OB mothers between 0 and 12 weeks. A higher prepregnancy BMI is a proxy for periconception overnutrition. In OW/OB mothers, levels of circulating glucose and fatty acids are high, and the foetus receives these excess amounts of glucose and fatty acids, which are metabolised and deposited as fat [35]. This is thought to be one of the associations that mediate the intergenerational cycle of obesity. Interestingly, some have noted that the infant–mother association of adiposity is limited only to girls [11,33,36]; however, due to our small sample size, we could not assess sex differences in infant body composition. Generally, females are identified with greater FM and less FFM than males throughout life, starting from infancy, and these differences have been attributed to higher testosterone levels in males that enhance the synthesis of protein and thereby promote the growth of FFM.

The weight status of mothers who participated in our study did not significantly differ from their prepregnancy parameters from 3 months postpartum. This could be associated with wide confidence intervals of the variables owing to the small sample size. In addition, the observed pattern, particularly the increase in weight and BMI from 9 to 12 months, may be due to random variation, and a larger sample is necessary to distinguish the change. Others have reported that a substantial proportion of women retain a higher BMI than their prepregnancy levels up to one year postpartum [37,38]. Elevated postpartum BMI can be a result of several factors, including dietary habits and physical activity [39]; however, women with higher prepregnancy BMI or gestational weight gain beyond recommended guidelines find it hard to return to their prepregnancy BMI within the first year postpartum [40,41]. While pregnancy-induced FM increase supports foetal growth and helps preparation for lactation, after parturition, increased caloric expenditure imposed by breastfeeding can contribute to a gradual loss of the gained weight over time [42]. It is estimated that lactation can result in an additional caloric expenditure of 300–500 calories per day, although this may vary based on the frequency and duration of breastfeeding [43]. Encouraging breastfeeding, along with tailored interventions such as nutrition counselling and physical activity guidance, can contribute to improved weight status and body composition outcomes during the postpartum, especially for women with OW/OB prepregnancy BMI.

Understanding infant–mother body composition correlations can help improve health outcomes for both mothers and infants. Positive associations between maternal and infant FM [33] have been observed in the early weeks of infant age [44]; however, studies exploring how associations change in the long term are lacking. Penfield-Cyr [8] showed that in obese women, maternal FM was negatively associated with infant FFM at 4 months postpartum, while maternal BMI was negatively associated with infant FFM at 7 months postpartum, suggesting that early life programming of obesity may be linked with FFM accrual contrary to FM. Further, Henry [45] found that the %FM in infants and mothers are not correlated at 2 years postpartum. However, in our study, infant and maternal %FM was significantly associated at 1 year postpartum after adjusting for maternal prepregnancy BMI. Despite the small sample size, our result warrants further study into the potential role of maternal body composition on infant growth patterns.

The strengths of our study include the use of reliable, validated body composition techniques, namely, ADP and deuterium dilution, and follow-up of the participants over a one-year period. The relatively small sample size and male-dominant gender distribution in the sample may reduce the generalisability of our results. We could not continue following up with Baby-bod study participants due to COVID-19-related restrictions in Tasmania during 2020. A potential limitation of this study is the reliance on self-reported preconception weight, which is a common approach in studies of this nature but may be associated with recall inaccuracies and social desirability bias. Additionally, weight measured using different scales could be associated with different measurement errors. Another limitation is the change of body composition technique for infants as PEA POD cannot accommodate infants aged over 6 months; however, previous research by others [21] and our team [46] have shown no significant between-method mean differences in measurements obtained from PEA POD and deuterium dilution technique. Due to the relatively small sample size, we deliberately avoided including many confounding variables, including infant feeding methods, in our analysis to prevent the risk of overfitting. Future research with larger sample sizes is needed to explore potentially relevant confounding factors such as maternal diet and infant feeding practices in greater detail.

## 5. Conclusions

This pilot study reveals that OW/OB maternal prepregnancy BMI is associated with increased infant %FM during 0–3 months of age, which can have lasting consequences for health throughout childhood and adulthood. In addition, our results underscore the significance of maternal prepregnancy BMI as a predictor of maternal postpartum weight status and body composition, and it controls the relationship between maternal and infant body composition until at least 12 months postpartum. These findings highlight the importance of a healthy BMI in the prepregnancy period for healthy body composition during the postpartum period and potentially long-term health outcomes in both infants and mothers. Nevertheless, further research is needed to validate and expand upon these findings. By implementing multifaceted strategies such as educating women about the importance of a healthy BMI, subsidising nutritious foods, creating supportive environments for physical activity, and encouraging regular prenatal and postpartum health check-ups, healthcare professionals and policymakers can better support women in achieving and maintaining a healthy BMI. 

## Figures and Tables

**Table 1:** Characteristics of the participants.

Variable	Mean ± SD or n/N (%)
***Mothers***	
Age at delivery (years)	29.89 ± 4.43
Caucasian ethnicity	27/29 (93.1%)
Prepregnancy weight (kg)	73.30 ± 15.50
Height (m)	1.65 ± 0.05
Prepregnancy BMI (kg/m^2^)	26.85 ± 5.59
Prepregnancy weight status: overweight/obese	18/29 (62%)
Primiparous pregnancy	14/29 (48.3%)
Caesarean delivery	8/29 (27.6%)
Gestational diabetes	3/29 (10.3%)
Gestation length (weeks)	39.9 ± 1.32
***Infants***	
Sex: male	22/29 (75.9%)
Birthweight (kg)	3.34 ± 0.45
Length at birth (cm)	49.69 ± 2.10
Continued breastfeeding until 12 months	16/25 (64%)

Values are mean ± standard deviation for continuous variables; n/N (%) number/total (percentage) for categorical variables.

**Table 2:** Associations between infant %FM at 12 months of age and maternal body composition variables.

Maternal Body Composition Variable	Unadjusted Analysis		Adjusted Analysis	
	β (95% CI)	*p*-Value	β (95% CI)	*p*-Value
Maternal %FM at 12 months postpartum	0.20 (−0.03, 0.45)	0.083	0.30 (0.01, 0.59)	0.040
Maternal prepregnancy BMI	−0.01 (−0.35, 0.37)	0.967	−0.24 (−0.65, 0.18)	0.251

%FM: percent fat mass; CI: confidence interval; adjusted analysis: maternal body composition variables adjusted for each other.

## Data Availability

The datasets used and/or analysed during the current study are available from the corresponding author upon reasonable request. The data are not publicly available due to privacy reasons.

## List of Abbreviations

ADP	air displacement plethysmography
BMI	body mass index
FFM	fat-free mass
FM	fat mass
%FM	percent fat mass
OW/OB	overweight/obesity
